# Antioxidant and antiradical activities depend on adrenal tumor type

**DOI:** 10.3389/fendo.2022.1011043

**Published:** 2022-09-30

**Authors:** Barbara Choromańska, Piotr Myśliwiec, Tomasz Kozłowski, Jerzy Łukaszewicz, Harelik Petr Vasilyevich, Jacek Dadan, Anna Zalewska, Mateusz Maciejczyk

**Affiliations:** ^1^ 1st Department of General and Endocrine Surgery, Medical University of Bialystok, Bialystok, Poland; ^2^ Department of General Surgery, Grodno State Medical University, Grodno, Belarus; ^3^ Experimental Dentistry Laboratory, Medical University of Bialystok, Bialystok, Poland; ^4^ Department of Hygiene, Epidemiology and Ergonomics, Medical University of Bialystok, Bialystok, Poland

**Keywords:** adrenal tumors, incidentaloma, pheochromocytoma, cushing’s/conn’s adenoma, antioxidants, total antiradical activity

## Abstract

The aim of the study was to assess the total antioxidant/oxidant status in the plasma and urine of patients with adrenal tumors. The study group consisted of 60 patients (31 women and 29 men) with adrenal masses, classified into three subgroups: non-functional incidentaloma, pheochromocytoma and Cushing’s/Conn’s adenoma. The number of patients was set *a priori* based on our previous experiment (α = 0.05, test power = 0.9). Antioxidant activity (Total Antioxidant Capacity (TAC), Total Oxidant Status (TOS), Oxidative Stress Index (OSI)) and antiradical activity (Radical-Scavenging Activity Assay (DPPH), Ferric-Reducing Antioxidant Power (FRAP)) were measured using colorimetric methods. FRAP level was decreased in plasma and urine incidentaloma *(p<0.0001)*, pheochromocytoma *(p<0.0001)* and Cushing’s/Conn’s adenoma *(p<0.0001)*, while DPPH antiradical activity only in plasma of patients with adrenal masses *(p<0.0001)*. Plasma TAC was increased in incidentaloma patients *(p=0.0192)*, whereas in pheochromocytoma group *(p=0.0343*) was decreased. Plasma and urine TOS *(p<0.0001)* and OSI *(p<0.01)* were significantly higher in patients with adrenal tumors. In pheochromocytoma patients, plasma and urine TAC *(p=0.001; p=0.002)*, as well as plasma plasma DPPH *(p=0.007)* and urine FRAP *(p=0.017)* correlated positively with normethanephrine. We are the first who showed reduced radical scavenging capacity in the plasma/urine of patients with adrenal masses. Nevertheless, plasma TAC was significantly higher in the incidentaloma group compared to controls. Therefore, plasma and urinary antioxidant and antiradical activities depend on the presence of the tumor. Lower levels of TAC, DPPH and FRAP clearly indicate a reduced ability to scavenge free radicals and thus a lack of effective protection against oxidative stress in patients with adrenal tumors. Both plasma and urine redox biomarkers can be used to assess systemic antioxidant status in adrenal tumor patients.

## Introduction

Although malignant adrenal tumors are rare, with 1-2 cases per 1 million people a year, benign adrenal masses are the most common of all tumors in humans (1). Typically, they are detected incidentally during diagnostic imaging due to other diseases, hence the term incidentaloma (2). Adrenal masses occur in up to 5% of the adult population, with malignancy rates in 1–12% (3). Even though, most of these masses are benign and nonfunctional, 1–15% may cause overproduction of hormones (aldosterone, cortisol or catecholamines) (3–5). Unfortunately, the pathogenesis of adrenal tumors is not fully understood. Currently, it is believed that most of them are caused by genetic abnormalities (6). Hypoxia-induced factor (HIF-1) deregulation has been involved in the pathogenesis of cancer secreting catecholamines (7). Indeed, the VHL/HIF axis mutation is most common in pheochromocytoma (8). Recent research has brought awareness to the key role of oxidative stress (OS) in cancer development (9–11). Reactive oxygen species (ROS) and HIF-1 interact with each other, intensifying the process of carcinogenesis under hypoxic conditions (12–14). In response to hypoxia, HIF-1 activation leads to an increased activity of NADPH oxidase, the main source of ROS in a cell (15). Overproduction of ROS disrupts cellular metabolism including many signaling pathways (NF-κB, PI3 kinase, MAPK or p21RAS) and induces oxidative damage to lipids and proteins (16, 17). The accumulated products of lipid and protein oxidation are cytotoxic increasing the structural and functional damage to cell organelles and inducing apoptosis (18). Further on, overproduction of ROS can damage nucleic acids and lead to cell death through necrosis (16). Therefore, aerobic organisms have developed a defense mechanism in the form of antioxidant barrier (19). Until now, little is known about the interaction of oxidants and antioxidants in the development of adrenal tumors. In our previous study, we have described abnormalities in both enzymatic and non-enzymatic antioxidant barrier (20). However, it is not known how the total antioxidant status changes in patients with adrenal tumors. The compounds with antioxidant properties can interact additively or synergistically with each other (21, 22). Therefore, total antioxidant capacity better characterizes the redox status of the biological system than the determination of individual antioxidants separately (23, 24). Therefore, the aim of this study was to evaluate the total antioxidant potential using various methods: total antioxidant capacity (TAC), iron reducing antioxidant power (FRAP) and the DPPH (2,2′-diphenyl-1-picrylhydrazyl) radical scavenging activity. Redox status was also assessed by measuring the total oxidant status (TOS) and the oxidative stress (OSI) index. Thus, the results of our study will provide an answer to the question: is the oxidation-reduction equilibrium shifted towards the oxidation?

Adrenal tumors may not show specific clinical symptoms and are usually detected accidentally. Due to their diversity, diagnostics are complicated and burdensome for the patient. Therefore, it is important to search new, more specific and sensitive markers in the material collected in a non-invasive manner. Importantly, the total antioxidant potential depending on the biological fluid (plasma, serum, urine, etc.). However, there are no studies characterizing the antioxidant status in different diagnostic biomaterials of patients with adrenal tumors. Therefore, the aim of our study was also a comparative evaluation of the total antioxidant capacity in the plasma and urine to assess their diagnostic utility.

## Materials and methods

The study was designed and conducted in accordance with the Guidelines for Good Clinical Practice and the Declaration of Helsinki. The study was also approved by the Bioethics Committee of the Medical University of Bialystok (code of permission: R-I-002/66/2015, APK.002.341.2020). All patients gave their informed consent to participate in this study.

The inclusion criterion for the study group was the presence of an adrenal tumor, while the control group included generally healthy subjects. The diagnosis of adrenal tumor was performed in the departments of internal diseases with an endocrine profile. The subjects from both study and control groups were qualified for the study based on a negative medical history concerning: neoplastic diseases, metabolic diseases (osteoporosis, gout, mucopolysaccharidosis, insulin resistance and type 1 diabetes), cardiovascular diseases, autoimmune diseases (ulcerative colitis, Hashimoto’s disease and Crohn’s disease), diseases of the genitourinary, digestive and respiratory systems, infectious diseases (HIV/AIDS, hepatitis A, B and C), acute inflammation, as well as pregnancy in women. The participants of the study were not abusing alcohol nor smoking. Additional exclusion criteria were taking nonsteroidal anti‐inflammatory drugs, glucocorticosteroids, antibiotics and antioxidant supplements (including iron preparations) for three months before collecting material for the study. Patients in all groups were on a diet (2000 kcal, including 55% carbohydrates, 30% fat, and 15% protein) determined by a dietician.

The study group consisted of 60 patients (31 women and 29 men aged from 50 to 65 years) with adrenal masses diameter > 4 cm and < 8 cm, who were treated using endoscopic adrenalectomy at the First Department of General and Endocrine Surgery at the University Hospital in Bialystok. The patients were classified into three subgroups: patients with non-functional incidentaloma (n=20), pheochromocytoma (n=20) and Cushing’s/Conn’s adenoma (n=20). In the adenoma subgroup Cushing’s syndrome was diagnosed in 11 patients and Conn’s syndrome in 9 patients. Preoperatively patients with Conn’s syndrome received potassium supplementation or spironolactone (aldosterone receptor blocker). Patients with phaeochromocytoma took doxazosin (a selective alpha-1-adrenergic receptor blocker) for 10 to 14 days before surgery to avoid intraoperative hypertensive crisis.

The control group included 60 healthy people (31 women and 29 men aged 50 to 65) whose blood counts and biochemical blood tests (Na^+^, K^+^, ALT, AST, creatinine and INR) were within the reference values. The subjects underwent abdominal ultrasound, which showed no abnormalities. The patients of the controls group were treated at the Specialist Dental Clinic at the Medical University of Bialystok.

The clinical and laboratory characteristics of the control and study groups are shown in **Table 1**.

**Table 1:** Clinical and routine laboratory characteristics of the controls, incidentaloma, pheochromocytoma, and Cushing’s/Conn’s adenoma patients.

	Controls*(n=60)*	Incidentaloma *(n=20)*	Pheochromocytoma *(n=20)*	Cushing’s/Conn’s adenoma *(n=20)*	ANOVA
**Age**	**58** ± 10	**59** ± 12	**57** ± 10	**58** ± 7	*p=0.908*
**Size of the tumor (cm)**	–	**4.053** ± 1.727	**3.889** ± 1.384	**3.685** ± 1.798	*p=0.7846*
**BMI (kg/m^2^)**	**23.16** ± 0.8042	**29.53** ± 4.97^***^	**27.58** ± 6.452^*^	**29.53** ± 3.554^****^	*p<0.0001*
**Na^+^ (mmol/l)**	**139.1** ± 2.149	**140.5** ± 2.503	**139.1** ± 2.516	**138.8** ± 2.579	*p=0.1015*
**K^+^ (mmol/l)**	**4.411** ± 0.3498	**4.489** ± 0.3129	**4.375** ± 0.351	**4.179** ± 0.5804	*p=0.0895*
**WBC (10^3^/μL)**	**7.33** ± 1.205	**7.349** ± 2.344	**7.675** ± 2.057	**7.596** ± 2.273	*p=0.926*
**RBC (10^6^/μL)**	**4.656** ± 0.3412	**4.816** ± 0.3703	**4.483** ± 0.5508	**4.545** ± 0.3733	*p=0.0799*
**HGB (g/dL)**	**13.62** ± 0.7923	**14.53** ± 1.195	**13.89** ± 1.48	**13.78** ± 1.138	*p=0.0585*
**PLT (10^3^/μL)**	**288.4** ± 14.08	**242.1** ± 69.22^**~^	**254.2** ± 49.86	**198.3** ± 46.7^****^^^	*p<0.0001*
**Glucose (mg/dL)**	**77.18** ± 6.372	**99.79** ± 21.88^***^	**91.56** ± 16.91^*^	**94.94** ± 19.14^**^	*p<0.0001*
**Aldosterone (ng/dL)**	**13.86** ± 7.062	**14.46** ± 8.45**^~^**	**17.4** ± 8.123	**23.2** ± 13.37^***^	*p=0.0008*
**Serum cortisol before** **10 a.m. (µg/dL)**	**12.19** ± 4.469	**15.43** ± 5.492	**14.04** ± 5.747	**16.88** ± 5.398^**^	*p=0.0019*
**Urine methanephrine (µg/24h)**	**146.5** ± 77.61	**103.7** ± 49.56**^^^^^^**	**727.5** ± 544.1^****^	**152.8** ± 82.38**^^^^^^**	*p<0.0001*
**Urine normethanephrine (µg/24h)**	**237.8** ± 83.04	**248.3** ± 123.7**^^^^^^**	**737.5** ± 292.9^****^	**362.2** ± 128.4^**^^^^^	*p<0.0001*

Results are presented as mean with standard deviation. *p<0.05, **p<0.01, ***p <0.001, ****p < 0.0001 indicate significant differences from the controls; ^^ p<0.01, ^^^^ p<0.0001 indicate significant differences from the pheochromocytoma group; ~ p<0.05 indicate significant differences from the Cushing’s/Conn’s group; body mass index (BMI), white blood cell count (WBC), red blood cell count (RBC), hemoglobin (HGB) and platelet count (PLT). Bold indicates mean values.

### Blood and rine collection

All samples from healthy individuals and patients with adrenal mass were collected in a fasting state. The patients declared, that they did not perform intense physical activity twenty-four hours prior to blood sampling. Blood samples were collected into EDTA and serum tubes (SARSTEDT, S-Monovette) and centrifuged at 4°C, 1789 x g for 10 minutes. The urine samples were collected in a sterile disposable container from the first-morning portion of urine from the middle stream immediately after bedtime and centrifuged at 252 x g for 5 minutes. In order to protect against oxidation, the supernatant was added (10 µl of 0.5 M BHT/1 ml of plasma/serum and urine) and stored at -80°C until appropriate determinations were made.

### Laboratory measurements

Serum cortisol before 10 a.m., serum aldosterone, Na^+^, K^+^, glucose, and urine methanephrine and normethanephrine, as well as full blood count were analyzed using an Abbott analyzer (Abbott Diagnostics, Wiesbaden, Germany).

### Redox assays

All reagents used to perform the redox assays were obtained from Sigma-Aldrich (Nümbrecht, Germany/Saint Louis, MO, USA). The absorbance of the samples was measured using Mindray MR-96 Microplate Reader (Mindray, Nanshan, China). Determinations of all tested parameters were carried out in triplicate samples. The results were standardized to 1 mg of total protein.

### Antioxidant/oxidant activity tests

#### Total antioxidant capacity

The level of plasma total antioxidant capacity (TAC) was determined using ABTS (2,2-azinobis-3-ethylbenzothiazoline-6-sulfonic acid) radical cation and Trolox (6-hydroxy-2,5,7,8-tetramethylchroman-2-carboxylic acid) as a standard. Absorbance was read spectrophotometrically at 660 nm (25).

#### Total oxidant status

In the presence of the oxidants contained in the sample, the level of plasma total oxidant status (TOS) was evaluated bichromatically at 560/800 nm based on the oxidation reaction of Fe^2+^ to Fe^3+^ (26).

#### Oxidative stress index (OSI)

Oxidative stress index (OSI) was counted as TOS to TAC ratio: OSI = TOS/TAC (27).

### Antiradical activity tests

#### Radical-scavenging activity assay (DPPH)

The antioxidant potential of plasma and urine was also assayed using DPPH (1,1-diphenyl-2- picrylhydrazyl) radical and Trolox as a standard (22). The absorbance of DPPH, after decolorization in the presence of antioxidants, was measured spectrophotometrically at 515 nm.

#### Ferric-reducing antioxidant power

The level of ferric-reducing antioxidant power (FRAP) was assayed using the reduction reaction of Fe^2+^ to Fe^3+^ an acidic environment. Absorbance of the resulting a colorful ferrous tripyridyltriazine (Fe^3+^-TPTZ) complex was measured colorimetrically at 592 nm (28).

### Hydrophilic antioxidants and hydrogen peroxide

#### Uric acid (UA)

UA concentration was analyzed using Abbott analyzer (Abbott Diagnostics, Wiesbaden, Germany).

#### Ascorbic acid

AA concentration was determined colorimetrically using Folin-Ciocalteu reagent. The absorption of the color product formed in the reaction between AA and Folin-Ciocalteu reagent was measured at 760 nm (29).

#### Albumin

The concentration of albumin was determined colorimetrically using bromocresol green solution. The absorbance was measured at 628 nm wavelength in the reaction between albumin and bromocresol green in succinate buffer.

#### The total phenolic content

TPC was assayed according to the Folin–Ciocalteu colorimetric method (30). The absorption was measured at 760 nm in the reaction between phenols and Folin–Ciocalteu reagent.

#### Total thiols

Total thiols concentration was measured colorimetrically at 420 nm using Ellman’s reagent (31). The concentration of thiol groups was counted on the basic of the calibration curve using reduced glutathione (GSH) as a standard.

#### Hydrogen peroxide

The concentration of H_2_O_2_ was measured using commercially available kit (The Amplex^®^ Red Hydrogen Peroxide/Peroxidase Assay Kit; Invitrogen, Molecular Probes, Paisley, United Kingdom) according to the manufacturer’s instructions. The H_2_O_2_ concentration was determined immediately after centrifuging the sample.

### Statistical analysis

Statistical analysis was performed using GraphPad Prism 7.0 (GraphPad Software, La Jolla, USA) and Microsoft Excel 16.49 for MacOS. The Shapiro–Wilk test were used to evaluate the distribution of the results and data were presented as mean ± SD. The homogeneity of variance was checked by Levine’s test. The groups were compared using one-way analysis of variance ANOVA with Tukey’s *post-hoc* test. Multiplicity adjusted p value was also calculated. Correlations between biomarkers and clinical parameters were assessed based on the Pearson correlation coefficient. Statistically significant value was p ≤ 0.05.

The number of patients was determined *a priori* based on the previous pilot study (*n* = 40). The power of the test was assumed as 0.9 and α = 0.05. Variables used for sample size calculation were plasma and urine TAC, TOS and FRAP. The ClinCalc online calculator provided the sample size for one group. The minimum number of patients was 17.

## Results

**Table 1** demonstrates a comparison of the clinical and laboratory characteristics of the controls and patients with adrenal masses: incidentaloma, pheochromocytoma, and Cushing’s/Conn’s adenoma. We found higher BMI values and serum glucose concentration in all study subgroups compared to the healthy controls. The PLT content was decreased in patients with incidentaloma and Cushing’s/Conn’s adenoma than in the controls and patients with pheochromocytoma. Urinary metanephrine and normetanephrine were increased in the pheochromocytoma group than the controls and incidentaloma and Cushing’s/Conn’s adenoma patients. However, concentration of serum cortisol and aldosterone were higher in Cushing’s/Conn’s adenoma group as compared to the controls.

### Plasma and urine concentration of total antioxidant capacity in patients with adrenal tumors

**Figure 1:**
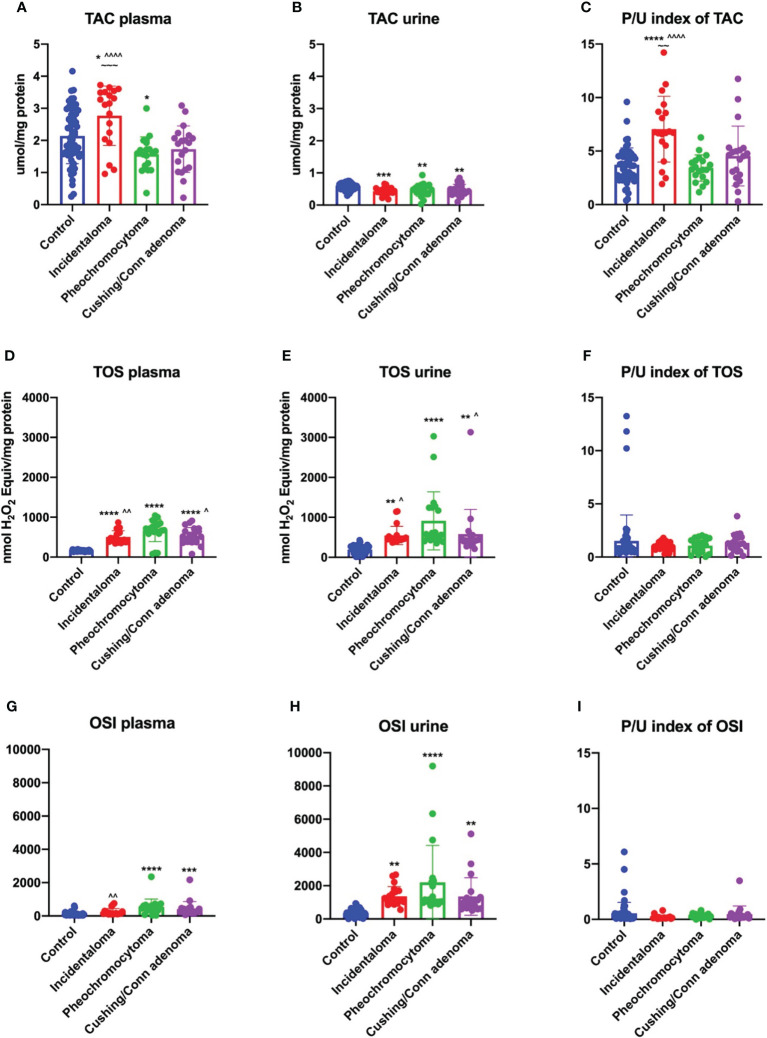
Plasma TAC **(A)**, TOS **(D)** and OSI **(G)**, urine TAC **(B)**, TOS **(E)** and OSI **(H)**, and plasma/urine index of TAC **(C)**, TOS **(F)** and OSI **(I)** of the controls, incidentaloma, pheochromocytoma, and Cushing’s/Conn’s adenoma patients. Results are presented as mean with standard deviation. *p<0.05, **p<0.01, ***p < 0.001, ****p < 0.0001 indicate significant differences from the controls; ^ p<0.05, ^^ p<0.01, ^^^^p<0.0001 indicate significant differences from the pheochromocytoma group; ~~ p<0.01, ~~~ p<0.001 indicate significant differences from the Cushing’s/Conn’s group; total antioxidant capacity (TAC), total oxidant status (TOS) and oxidative status index (OSI).

The TAC test is used to assess total antioxidant activity, especially non-enzymatic antioxidant activity. The TAC test determines the activity of known and unknown antioxidants and detects the synergism between the antioxidants (32, 33). Interestingly, plasma TAC was increased in incidentaloma patients (+29%, *p=0.0192*), whereas in pheochromocytoma group was decreased (-27%, *p=0.0343*) as compared with the controls. Additionally, plasma TAC was greater in incidentaloma group (+77%, *p<0.0001*; +60%, *p=0.0006*; respectively) than the pheochromocytoma and Cushing’s/Conn’s adenoma patients (**Figure 1A**). In urine TAC values was significantly diminished in patients with adrenal masses: incidentaloma (-27%, *p=0.0001*), pheochromocytoma (-20%, *p=0.0063*) and Cushing’s/Conn’s adenoma (-21%, *p=0.0037*) in comparison with the controls (**Figure 1B**). However, plasma/urine index of TAC was enhanced only in incidentaloma patients (+90%, *p<0.0001*; +101%, *p<0.0001*; +55%, *p=0.0015*; respectively) than the controls, pheochromocytoma and Cushing’s/Conn’s adenoma (**Figure 1C**).

### Plasma and urine concentration of total oxidant status in patients with adrenal tumors

TOS is indicator to determine all oxidants in the sample, which can more specifically reflect the changes of oxidant capacity than various oxidants measured separately. We found significantly higher values of plasma and urine TOS in all study subgroups: incidentaloma (+214%, *p<0.0001*; +184%, *p<0.0001*), pheochromocytoma (+313%, *p<0.0001*; +375%, *p<0.0001*) and Cushing’s/Conn’s adenoma (+229%, *p<0.0001*; +200%, *p<0.0001*) than the healthy controls. Moreover, plasma TOS was increased in pheochromocytoma (+31%, *p=0.0082*; +26%, *p=0.0336*; respectively) than the incidentaloma and Cushing’s/Conn’s adenoma. Similarly to plasma, in urine patients with pheochromocytoma (+67%, *p=0.0245*; +58%, *p=0.0462*; respectively) had greater value than the incidentaloma and Cushing’s/Conn’s adenoma patients (**Figures 1D, E**). Plasma/urine index of TOC did not differ between study groups (**Figure 1F**).

### Plasma and urine oxidative stress index in patients with adrenal tumors

We noticed increased OSI in plasma of pheochromocytoma (+421%, *p<0.0001*) and Cushing’s/Conn’s adenoma (+304%, *p=0.0003*) patients as compared to the healthy controls. Further on, plasma OSI was greater in pheochromocytoma (+123%, *p=0.009*) than incidentaloma patients (**Figure 1G**). In urine OSI was enhanced in all study subgroups: incidentaloma (+292%, *p=0.0012*), pheochromocytoma (+533%, *p<0.0001*) and Cushing’s/Conn’s adenoma (+288%, *p=0.0015*) in comparison with the controls (**Figure 1H**). We did not find any differences in plasma/urine index of OSI in study groups (**Figure 1I**).

### Plasma and urine antiradical activity in patient with adrenal tumors

**Figure 2:**
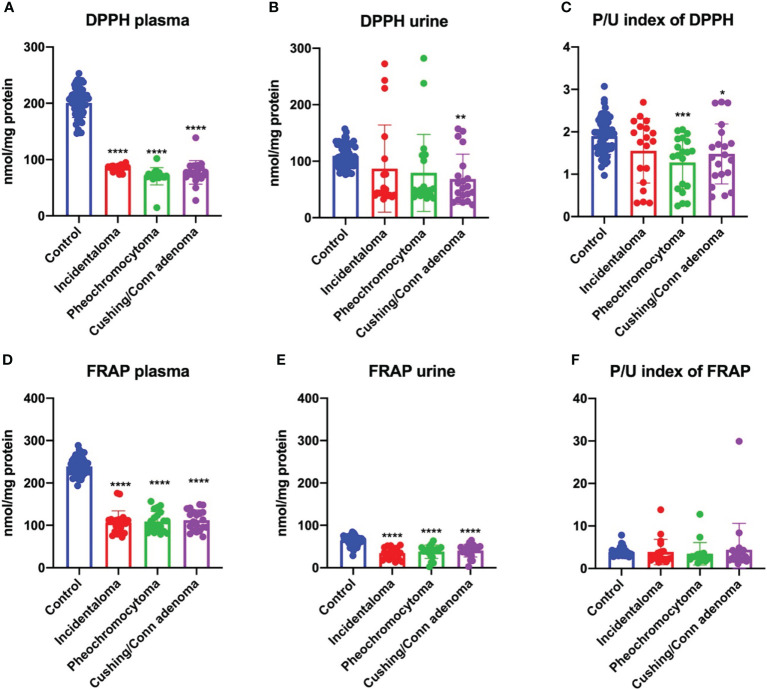
Plasma DPPH **(A)** and FRAP **(D)**, urine DPPH **(B)** and FRAP **(E)**, and plasma/urine index of DPPH **(C)** and FRAP **(F)** of the controls, incidentaloma, pheochromocytoma, and Cushing’s/Conn’s adenoma patients. Results are presented as mean with standard deviation. *p<0.05, **p<0.01, ***p < 0.001, ****p < 0.0001 indicate significant differences from the controls; 2,2′-diphenyl-1-picrylhydrazyl radical (DPPH) and ferric reducing antioxidant power (FRAP).

The antiradical activity is usually determined by measuring the scavenging of the synthetic radical 2,2′-diphenyl-1-picrylhydrazyl (DPPH). It is also measured in ferric reducing antioxidant power (FRAP) assay. The reduction assays assume that antioxidants are reducing agents which react with free radicals (33, 34). We observed markedly lower plasma DPPH in all patients with adrenal masses: incidentaloma (-57%, *p<0.0001*), pheochromocytoma (-65%, *p<0.0001*) and Cushing’s/Conn’s adenoma (-61%, *p<0.0001*) than the controls (**Figure 2A**). Whereas, in urine, DPPH was decreased only in Cushing’s/Conn’s adenoma patients (-37%, *p=0.0056*) as compared to the controls (**Figure 2B**). However, plasma/urine index of DPPH was diminished in pheochromocytoma (-33%, *p=0.0003*) and Cushing’s/Conn’s adenoma (-22%, *p=0.0289*) in comparison with the controls (**Figure 2C**).

The plasma and urine FRAP was significantly decreased in all study subgroups: incidentaloma (-55%, *p<0.0001*; -46%, *p<0.0001*), pheochromocytoma (-54%, *p<0.0001*; -41%, *p<0.0001*) and Cushing’s/Conn’s adenoma (-53%, *p<0.0001*; -37%, *p<0.0001*) as compared to the controls (**Figures 2D, E**). However, there were no differences between the study groups in the plasma/urine index of FRAP (**Figure 2F**).

### Serum/plasma and urine concentration of selected antioxidants and hydrogen peroxide

**Table 2** presents levels of selected antioxidants and hydrogen peroxide.

**Table 2:** The serum/plasma and urine concentration of selected antioxidants and hydrogen peroxide of the controls, incidentaloma, pheochromocytoma, and Cushing’s/Conn’s adenoma patients.

	Controls*(n=60)*	Incidentaloma*(n=20)*	Pheochromocytoma *(n=20)*	Cushing’s/Conn’s adenoma *(n=20)*	ANOVA
**Serum/plasma**					
**UA (mg/dL)**	**5.427** ± 1.045	**4.393** ± 1.083^**^	**5.309** ± 1.187	**5.114** ± 1.225	*p=0.011*
**AA (µg/mg protein)**	**17.03** ± 3.535	**14.98** ± 2.498^*^	**14.34** ± 1.46^**^	**14.37** ± 1.733^**^	*p=0.0001*
**Albumin (mg/dL)**	**4.934** ± 0.943	**4.223** ± 1.107^*^	**4.24** ± 0.568^*^	**4.138** ± 0.896^**^	*p=0.0005*
**TPC (µg/mg protein)**	**66.23** ± 27.25	**38.13** ± 24.51^**^	**52.41** ± 34.65	**49.05** ± 34.21^**^	*p=0.0032*
**Total thiols** **(µg/mg protein)**	**21.07** ± 4.564	**26.47** ± 8.702^*^^^^~~^	**16.07** ± 7.904^*^	**18.66** ± 9.837	*p<0.0001*
**H_2_O_2_ (pmol/µL)**	**6.319** ± 1.25	**16.96** ± 5.731^****^^^^~~~~^	**10.14** ± 4.372^***^	**9.344** ± 4.765^**^	*p<0.0001*
**Urine**					
**UA (mg/dL)**	**54.63** ± 10.47	**40.95** ± 7.78^****^	**41.26** ± 7.577^****^	**38.95** ± 10.71^****^	*p<0.0001*
**AA (µg/mg protein)**	**17.02** ± 5.294	**13.71** ± 5.902	**12.54** ± 6.243^*^	**12.42** ± 4.913^**^	*p=0.0009*
**Albumin** **(µg/mg protein)**	**19.87** ± 5.095	**19.76** ± 8.811	**19.93** ± 6.843	**21.47** ± 7.835	*p=0,8016*
**TPC (µg/mg protein)**	**214.2** ± 61.04	**165.6** ± 74.57^*^	**175.3** ± 71.74	**190.9** ± 71.1	*p=0.0171*
**Total thiols** **(µg/mg protein)**	**1.026** ± 0.4782	**0.9718** ± 0.5693	**0.9658** ± 0.5305	**0.9365** ± 0.465	*p=0.895*
**H_2_O_2_ (pmol/mg protein)**	**0.4988** ± 0.0791	**0.699** ± 0.053^****^	**0.606** ± 0.102^**^	**0.6283** ± 0.247^***^	*p<0.0001*

Results are presented as mean with standard deviation. * p<0.05, **p<0.01, *** p <0.001, **** p < 0.0001 indicate significant differences from the controls; ^^^^ p<0.0001 indicate significant differences from the pheochromocytoma group; ~~ p<0.01, ~~~~ p<0.0001 indicate significant differences from the Cushing’s/Conn’s group; hydrogen peroxide (H_2_O_2_), ascorbic acid (AA), Total Phenolic Content (TPC) and uric acid (UA). Bold indicates mean values.

We found diminished concentration of UA only in plasma of incidentaloma patients (-19%, *p=0.0056*), while in urine the UA concentration was lower in all study groups: incidentaloma (-25%, *p<0.0001*), pheochromocytoma (-24%, *p<0.0001*) and Cushing’s/Conn’s adenoma (-29%, *p<0.0001*) in comparison with the controls.

The plasma content of AA was decreased in patients with adrenal incidentaloma (-12%, *p=0.0335*), pheochromocytoma (-16%, *p=0.0031*) and Cushing’s/Conn’s adenoma (-16%, *p=0.0037*), whereas in urine content of AA was lower in pheochromocytoma (-26%, *p=0.011*) and Cushing’s/Conn’s adenoma (-27%, *p=0.0085*) than the controls.

We observed decreased concentration of serum albumin in patients with adrenal masses: incidentaloma (-16%, *p=0.0168*), pheochromocytoma (-16%, *p=0.0206*) and Cushing’s/Conn’s adenoma (-14%, *p=0.0068*) than the controls.

The plasma content of total thiols in patients with incidentaloma was increased (+26%, *p=0.018*) while in pheochromocytoma was diminished (-24%, *p=0.0331*) as compared to the controls. Additionally, patients with incidentaloma had higher plasma total thiols content than pheochromocytoma (+65%, *p<0.0001*) and Cushing’s/Conn’s adenoma (+42%, *p=0.0033*) groups.

Patients with incidentaloma had lower content of serum and urine (-42%, *p=0.0034*; -23%, *p=0.029*) TPC than the controls, whereas Cushing’s/Conn’s adenoma had decreased only in serum.

We observed increased concentration of H_2_O_2_ in plasma and urine of patients with adrenal masses: incidentaloma (+168%, *p<0.0001*; +40%, *p<0.0001*), pheochromocytoma (+60%, *p=0.0004*; +21%, *p=0.0058*) and Cushing’s/Conn’s adenoma (+48%, *p=0.0082*; +26%, *p=0.0005*) than the controls. Interestingly, plasma concentration of H_2_O_2_ was greater in incidentaloma patients than pheochromocytoma (+36%, *p<0.0001*) and Cushing’s/Conn’s adenoma (+82%, *p<0.0001*) groups.

### Correlations between the analyzed redox biomarkers and clinical parameters in the controls

**Figure 3:**
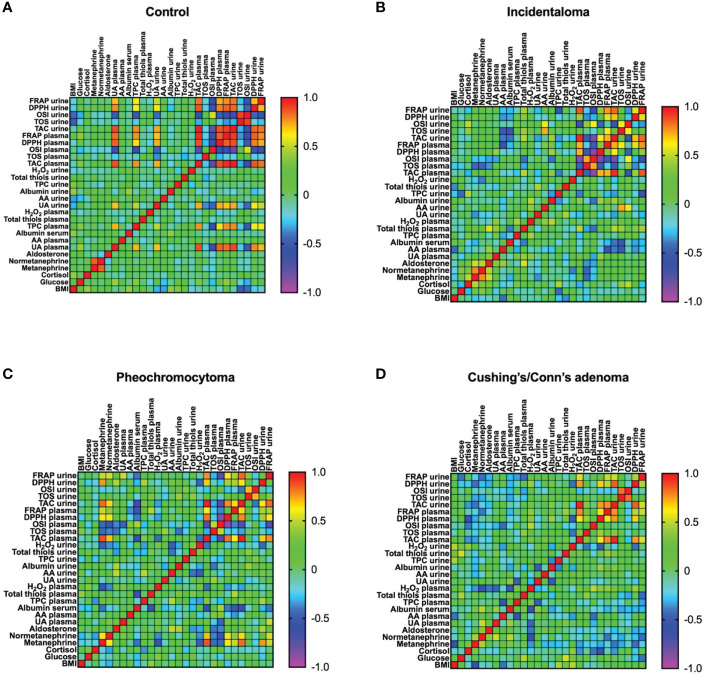
Correlations between the analyzed redox biomarkers and clinical parameters in in plasma and urine of the controls **(A)** and patients with incidentaloma **(B)**, pheochromocytoma **(C)**, and Cushing’s/Conn’s adenoma **(D)**. Ferric reducing antioxidant power (FRAP), 2,2′-diphenyl-1-picrylhydrazyl radical (DPPH), oxidative status index (OSI), total oxidant status (TOS), total antioxidant capacity (TAC), body mass index (BMI).

In the controls, plasma TAC correlated highly positively with urine TAC *(R=0.981*, *p<0.0001*), plasma DPPH *(R=0.886*, *p<0.0001*) and plasma FRAP *(R=0.945, p<0.0001)*, as well as negatively with plasma OSI *(R=-0.724, p<0.0001)*. Plasma OSI was associated positively with urine OSI *(R=0.541, p<0.0001)*, and negatively with plasma DPPH *(R=-0.573, p<0.0001)* and plasma FRAP *(R=-0.604, p<0.0001)*. The positive associations were between plasma DPPH and plasma FRAP *(R=0.858, p<0.0001)*, plasma DPPH and urine DPPH *(R=0.797, p<0.0001)*, plasma FRAP and urine FRAP *(R=0.775, p<0.0001)*, urine TAC and urine DPPH *(R=0.838, P<0.0001)*, urine TAC and urine FRAP *(R=0.812, p<0.0001)*, urine TOS and urine OSI *(R=0.904, p<0.0001)*, and urine DPPH and urine FRAP *(R=0.702, p<0.0001)*. We observed negative correlation between plasma TOS and plasma DPPH *(R=-0.291, p=0.024)*, urine TAC and urine OSI *(R=-0.63, p<0.0001)*, urine TAC and urine TOS *(R=-0.308, p=0.017)*, urine TOS and urine FRAP *(R=-0.27, p=0.037)*, urine OSI and urine DPPH *(R=-0.444, p<0.0001)*, as well as urine OSI and urine FRAP *(R=-0.52, p<0.0001)*. Moreover, BMI correlated positively with cortisol *(R=0.415, p=0.023)*, and negatively with urine TOS *(R=-0.481, p=0.007)* and urine OSI *(R=-0.435, p=0.016)*. Plasma FRAP was positively associated with glucose *(R=0.4, p=0.035)*, while normethanephrine correlated positively with cortisol *(R=0.282, p=0.029)* and methanephrine *(R=0.854, p<0.0001)*. Plasma UA correlated positively with plasma TPC *(R=0.713, p<0.0001)*, plasma and urine TAC *(R=0.879, p<0.0001; R=0.889, p<0.0001)*, plasma and urine DDPH *(R=0.852, p<0.0001; R=0.779, p<0.0001)*, plasma and urine FRAP *(R=0.832, p<0.0001; R=0.705, p<0.0001)*, and negatively with plasma and urine OSI *(R=-0.608, p<0.0001; R=-0.504, p<0.0001)*. Plasma TPC was associated positively with TAC *(R=0.77, p<0.0001; R=0.779, p<0.0001)*, DDPH *(R=0.645, p<0.0001; R=0.618, p<0.0001)*, FRAP *(R=0.772, p<0.0001; R=0.632, p<0.0001)*, and negatively with plasma and urine OSI *(R=-0.6, p<0.0001; R=-0.506)*. Urine UA positively correlated with plasma UA *(R=0.691, p<0.0001)* and plasma TPC *(R=0.495, p<0.0001)*. We observed positive correlation between plasma TPC and plasma UA *(R=0.713, p <0.0001)* and glucose *(R=0.382, p=0.045)*. The urine albumin was positively associated with plasma albumin *(R=0.412, p=0.001)* and BMI *(R=0.415, p=0.023)*. (**Figure 3A**).

### Correlations between the analyzed redox biomarkers and clinical parameters in incidentaloma patients

In incidentaloma patients, plasma TAC was associated positively with plasma DPPH *(R=0.712, p=0.001)*, plasma FRAP *(R=0.714, p=0.001)* and urine TAC *(R=0.943, p<0.0001)*, as well as negatively with plasma OSI *(R=-0.866. p<0.0001)*. The positive correlations were between plasma TOS and plasma OSI *(R=0.733, p<0.0001)*, plasma OSI and urine OSI *(R=0.613, p=0.005)*, plasma FRAP and urine FRAP *(R=0.759, p<0.0001)*, urine TAC and urine DPPH *(R=0.697, p=0.001)*, urine TAC and urine FRAP *(R=0.84, p<0.0001)*, urine TOS and urine OSI *(R=0.594, p=0.006)*, as well as urine DPHA and urine FRAP *(R=0.563, p=0.015)*. Whereas. we found negative associations between plasma TOS and plasma DPPH *(R=-0.498, p=0.025)*, plasma OSI and plasma DPPH *(R=-0.844, p<0.0001)*, plasma OSI and plasma FRAP *(R=-0.493, p=0.032)*, as well as urine TAC and urine OSI *(R=-0.553, p=0.011)*. Further on, plasma TOS negatively correlated with normethanephrine *(R=-0.448, p=0.048)*, whereas urine TOS wit serum albumin *(R=-0.519, p=0.019)*. We observed positive correlations between cortisol and methanephrine *(R=0.485, p=0.03)*, cortisol and urine OSI *(R=0.496, p=0.026)*, methanephrine and normethanephrine *(R=0.781, p<0.0001)*, methanephrine and aldosterone *(R=0.529, p=0.017)*, as well as normethanephrine and aldosterone *(R=0.483, p=0.031)*. Plasma total thiols was positively associated with plasma and urine TAC *(R=0.484, p=0.036; R= 0.482, p=0.032)* as well as with aldosterone *(R=0.549, p=0.12)*, whereas urine total thiols correlated with urine UA *(R=0.464, p=0.039)*. We found negative correlation between plasma AA and plasma FRAP *(R=-0.553, p=0.012)* and urine TOS *(R=-0.501, p=0.024)*. Urine AA was positively associated with TOS *(R=0.722, p<0.0001)* and OSI (R=0.589, p=0.006) in urine. Urine TPC negatively correlated with urine FRAP *(R=-0.552, p=0.014)*. Plasma H_2_O_2_ was associated positively with urine albumin *(R=0.586, p=0.03)* (**Figure 3B**).

### Correlations between the analyzed redox biomarkers and clinical parameters in pheochromocytoma patients

In pheochromocytoma subgroup, plasma TAC correlated positively with plasma DPPH *(R=0.815, p<0.0001)*, plasma FRAP *(R=0.61, p=0.004)* and urine TAC *(R=0.973, p<0.0001)*, whereas negatively plasma OSI *(R=-0.711, p<0.0001)*. We found positive associations between plasma TOS and plasma OSI *(R=0.494, p=0.027)*, plasma DPPH and urine DPPH *(R=0.451, p=0.046)*, plasma FRAP and urine FRAP *(R=0.458, p=0.049)*, urine TAC and urine DPPH *(R=0.639, p=0.002)*, urine TAC and urine FRAP *(R=0.835, p<0.0001)*. Methanephrine was associated positively with normethanephrine *(R=0.631, p=0.003)*, plasma and urine TAC *(R=0.877, p<0.0001; R=0.83, p<0.0001)*, as well as negatively with urine H_2_O_2_ (*R=-0.474, p=0.035)*, DPPH *(R=0.633, p=0.003; R=0.76, p<0.0001)*, and FRAP *(R=0.511, p=0.021; R=0.515, p=0.024)*. We observed that normethanephrine correlated positively with plasma and urine TAC *(R=0.698, p=0.001; R=0.657, p=0.002)*, plasma DPPH *(R=0.586, p=0.007)* and urine FRAP *(R=0.54, p=0.017)*, as well as negatively with plasma OSI *(R=-0.525, p=0.017)*. The negative correlation was also between plasma OSI and plasma DPPH *(R=-0.869, p<0.0001)*. Plasma UA was positively associated with urine FRAP *(R=0.521, p=0.027)* and negatively with plasma OSI *(R=-0.465, p=0.045)*. We observed positive correlation between plasma TPC and urine DPPH *(R=0.462, p=0.047)*. Plasma total thiols was negatively associated with serum albumin *(R=-0.553, p=0.011)*. We found positive correlation between urine AA and aldosterone *(R=0.503, p=0.033)* (**Figure 3C**).

### Correlations between the analyzed redox biomarkers and clinical parameters in Cushing’s/Conn’s adenoma patients

In Cushing’s/Conn’s adenoma patients, plasma TAC highly positively correlated with plasma DPPH *(R=0.637, p=0.002)*, plasma FRAP *(R=0.782, p<0.0001)* and urine TAC *(R=0.956, p<0.0001)*. The positive correlations were between plasma DPPH and plasma FRAP *(R=0.58, p=0.007)*, plasma DPPH and urine DPPH *(R=0.674, p=0.001)*, plasma FRAP and urine FRAP *(R=0.516, p=0.02)*, urine TAC and urine DPPH *(R=0.852, p<0.0001)*, urine TAC and urine FRAP *(R=0.527, p=0.017)*, urine TOS and urine OSI *(R=0.821, p<0.0001)*, and urine DPPH and urine FRAP *(R=0.469, p=0.037)*. Additionally, plasma DPPH was associated negatively with cortisol *(R=-0.569, p=0.009)*, whereas methanephrine positively with normethanephrine *(R=0.457, p=0.043)*. We found positive correlation between plasma UA and normethanephrine *(R=0.522, p=0.018)*. Plasma and urine total thiols correlated positively with glucose *(R=0.468, p=0.05; R=0.535, p=0.022)*. The positive correlations were between plasma serum albumin and normethanephrine *(R=0.486, p=0.035)*, as well as urine TPC and BMI *(R=0.461, p=0.041)*, whereas negative correlations were between plasma TPC and plasma AA *(R=-0.542, p=0.017*), as well as urine albumin and urine UA *(R=-0.49, p=0.028)*. Plasma H_2_O_2_ was negatively associated with normethanephrine *(R=-0.541, p=0.014)*, plasma UA *(R=-0.45, p=0.046)* and plasma albumin *(R=-0.534, p=0.019)*, while urine H_2_O_2_ positively associated with BMI *(R=0.477, p=0.033)*. (**Figure 3D**).

## Discussion

In recent years, many studies have been conducted trying to explain the pathogenesis of cancer. The burden of cancer continues to increase worldwide. According to the World Health Organization (WHO), cancer is the second leading cause of death in the world, accounting for approximately 9.6 million deaths in 2018 (35). Numerous studies suggest that redox imbalance may be a factor predisposing to cancer development (9, 36, 37). However, it has not yet been clarified in what direction the redox equilibrium is shifted and whether these disorders may be involved in the development of adrenal tumors. This is the first study to evaluate the total antioxidant potential in patients with adrenal tumors. Additionally, we compared redox status depending on the type of the tumor: incidentaloma, pheochromocytoma and Cushing’s/Conn’s adenoma.

Antioxidants can act additively or synergistically, and can be absorbed and utilized in the body in different ways (38). Therefore, the assessment of total antioxidant activity provides more reliable information about the biological system than the assessment of individual antioxidants separately (21). There are many different methods for measuring total antioxidant activity. The contribution of individual antioxidants varies, because the same antioxidants have different reactivity in various methods (39, 40). Moreover, in order to correctly measure total antioxidant activity, it is recommended to perform at least two different tests. These methods use the ability of the test compound or product to scavenge free radicals and/or metal ions involved in the oxidation reaction.

It is also important to distinguish between antioxidant and antiradical activity. Antioxidant activity is characterized by the ability to inhibit the oxidation process, while antiradical activity is the ability to react with free radicals (41). In our study, we demonstrated reduced radical scavenging capacity in patients with incidentaloma (↓DPPH and ↓FRAP in plasma), pheochromocytoma (↓DPPH in plasma, ↓FRAP in plasma and urine) and Cushing’s/Conn adenoma (↓DPPH and ↓FRAP in plasma and urine) compared to the control group. Antioxidant capacity assessed in the TAC assay was also decreased in the plasma of pheochromocytoma patients and in the urine of all patients with adrenal masses. Nevertheless, plasma TAC levels were also significantly higher in the incidentaloma group compared to controls.

The DPPH test uses stable 1,1-diphenyl-2- picrylhydrazyl free radical and thus reflects the radical scavenging process and antiradical activity (33). The FRAP method is based on the reduction of iron ions by antioxidants contained in the sample (42). The contribution of individual antioxidants to the total antioxidant potential varies depending on the test used. Due to low pH = 3.6, share of GSH and thiol groups in the total antioxidant potential is significantly lower in the FRAP assay than in DPPH and TAC methods (22, 28). Therefore, plasma FRAP much better reflect the antioxidant potential of the human body (38).

In our study, we observed decreased plasma DPPH and FRAP in all study groups: incidentaloma, pheochromocytoma and Cushing’s/Conn’s adenoma. This suggests depletion of antioxidant reserves and/or increased free radical production in patients with adrenal masses. This is confirmed by the decrease in blood hydrophilic antioxidants such as AA and albumin compared to controls. TPC content was also significantly reduced in patients with incidentaloma and Cushing’s/Conn’s adenoma. In contrast, in urine we found a decrease in UA, the most important of the antioxidants in this biological fluid. Urinary AA concentrations were also significantly lower in patients with pheochromocytoma and Cushing’s/Conn’s adenoma. Although we did not directly evaluate the rate of ROS production in our patients, total oxidative potential (TOS) was significantly higher in adrenal cancer cases as compared to heathy controls. This parameter expresses the total oxidant content in the biological material and may indicate increased ROS formation in patients with adrenal masses. Importantly, this biomarker indicates an increase in both free-radical and non-radical oxidant species.

TAC measures only part of the antioxidant capacity, i.e. non-enzymatic activity, and is mainly influenced by antioxidants present at the highest concentrations. Uric acid and thiol protein groups have the largest share in TAC in human plasma (32). Therefore, the higher plasma TAC in incidentaloma patients may be due to an increased pool of circulating thiols. Indeed, total thiol levels were significantly higher only in patients with incidentaloma. This can be also evidenced by the correlations we found in our study. Plasma thiols was positively associated with plasma and urine TAC *(R=0.484, p=0.036; R= 0.482, p=0.032)* as well as with aldosterone *(R=0.549, p=0.12).* It also suggests an adaptive response to overproduction of ROS. The strengthening of the antioxidant barrier is the primary protective mechanisms against systemic oxidative stress. In this study, in addition to TOS, we also evaluated hydrogen peroxide production. H_2_O_2_ is not a free radical. Nevertheless, it has the ability to migrate through cell membranes and, in high concentrations, exhibits a strong cytotoxic effects. Hydrogen peroxide levels were significantly higher in the plasma of incidentaloma group compared to patients with other adrenal masses and healthy controls.

However, we also observed diminished plasma TAC in patients with pheochromocytoma. This may be a result of decreased plasma concentration of GSH, the major non-enzymatic antioxidant in these patients (20). Lower GSH concentrations lead to the intensification of the inflammatory process with an increase in the secretion of inflammatory mediators: IL-1β and TNF-α. Depletion of glutathione reserves also promotes oxidative and carbonyl stress, which may be responsible for the development of metabolic complications of adrenal tumors. However, further studies are needed to clarify the sources of increased ROS production in adrenal tumor cases.

The question now arises: is there a shift in redox equilibrium in favor of oxidation reactions? For this purpose, we calculated the oxidative stress index (OSI), which is the quotient of total antioxidant potential (TAC) to TOS. OSI was significantly higher in all patients with adrenal tumors and therefor antioxidant/oxidant barrier is shifted towards an increased oxidation process. Thus, in patients with adrenal tumors, oxidative damage to proteins, lipids, and DNA may be exacerbated. Although we observed disturbances in the redox homeostasis in all study groups, they were the most severe in patients with pheochromocytoma. Increased oxidative stress in patients with phaeochromocytoma can be associated with HIF-1 (hypoxia-inducible factor 1) activity. Under hypoxic conditions, HIF-1 by stabilizing HIF-1α, increases the activity of NADPH oxidase, contributing to the ROS overproduction. Moreover, most patients with adrenal gland tumors are overweight or obese. It is well known that an excessive amount of adipose tissue leads to increased production of ROS (43). Therefore, the question arises whether the redox disturbances are not the result of increased body weight or metabolic disorders of obesity. Although we have not investigated this directly, it can be speculated that the increased oxidative stress in patients with adrenal tumors may be associated with obesity. It has been described that adipokines secreted by adipose tissue can activate nuclear factor kappa B (NF-κB), which induces the secretion of proinflammatory cytokines (IL-1. IL-6. IL-8), tumor necrosis factor α (TNF-α), as well as impairs the bioavailability of NO and increases the formation of free radicals (43–46). Further on, patients with functional adrenal tumors, especially phaeochromocytomas suffer often from impaired lipid and glucose metabolism, and insulin resistance (47), which may be the result of increased production of catecholamines, obesity, as well as the advantage of the oxidative process over antioxidant (48). In addition, increased cortisol secretion can exacerbate oxidative stress. Although cortisol enhances the process of gluconeogenesis, it also enhances lipolysis of adipose tissue, leading to hyperglycemia and hyperlipidemia (49–51). Thus, we cannot exclude that metabolic abnormalities lead to impaired redox homeostasis in patients with adrenal tumors. Nevertheless, our patients had an adequate glucose profile, indicating that impaired redox balance depends on the presence of the tumor.

It should also be noted that the total antioxidant potential may vary depending on the biological fluid in which it is measured. Parameters that assess redox homeostasis are usually measured in serum or plasma as a stable environment for systemic biomarkers (52). Nevertheless, Il’yasova et al. (53) argue that urine is a better biological fluid for the evaluation of oxidative stress markers than plasma or serum; and urinary oxidative stress parameters may reflect local and systemic oxidative status (52). The urine has a lower content of metals and ROS promoters, therefore in the urine there is a lower risk of obtaining results with elevated values of oxidative stress markers (53). In this study we observed higher TAC, DPPH and FRAP values in the plasma than in urine. However, it was also observed that urine TAC had similar or higher values than in blood plasma (34). Therefore, it is important to check whether redox biomarkers correlate between different body fluids. Antioxidant status measured in body fluids generally reflect a local, not a systemic, redox homeostasis (54). However, we found positive correlations between plasma FRAP and urine FRAP in patients with incidentaloma. In pheochromocytoma subgroup, plasma TAC correlated positively with urine TAC, as well as plasma DPPH and urine DPPH, plasma FRAP and urine FRAP. In Cushing’s/Conn’s adenoma, plasma TAC highly positively correlated with urine TAC, plasma DPPH with urine DPPH and plasma FRAP with urine FRAP. This indicates that urinary antioxidant status reflects changes in blood and can be used to assess systemic redox imbalances. These hypotheses are also supported by the correlations between plasma/urinary antioxidant status and the classical biomarkers evaluated to assess disease progression: cortisol, metanephrine, and normetanephrine.

In the study groups, both TAC, DPPH, and FRAP generally did not correlate with UA concentration and total polyphenolic content. Thus, as opposed to healthy people, these compounds may be marginally responsible for plasma/urine antioxidant activity. The weakening of the antioxidant barrier may be due to depletion of other low molecular weight antioxidants such as lipophilic α-tocopherol, β-carotene, retinol, and coenzyme Q10. This issue requires further research and may be of great clinical importance.

Our study confirms previous reports that patients with adrenal tumors are especially vulnerable to oxidative stress and oxidative damage (20, 55). Although our study does not explain this, antioxidant supplementation may be considered in patients with adrenal tumors. Clinical trials evaluating the utility of antioxidant therapy/dietary modifications in patients with adrenal masses are needed. Studies to elucidate the reasons for impaired redox homeostasis in these patients are also essential.

Summarizing, we demonstrated reduced radical scavenging capacity in the plasma/urine of patients with adrenal masses. Nevertheless, plasma TAC was significantly higher in the incidentaloma group compared to controls. Thus, plasma and urinary antioxidant and antiradical activities depend on the presence of the tumor. Both plasma and urine redox biomarkers can be used to assess systemic antioxidant status in adrenal tumor patients. Although antioxidants are the main defense mechanism against ROS overproduction, the reduced levels of TAC, DPPH and FRAP clearly indicate a reduced ability to scavenge free radicals and thus a lack of effective protection against oxidative stress in patients with adrenal tumors.

## Data availability statement

The raw data supporting the conclusions of this article will be made available by the authors, without undue reservation.

## Ethics statement

The studies involving human participants were reviewed and approved by Bioethics Committee of the Medical University of Bialystok. The patients/participants provided their written informed consent to participate in this study.

## Author contributions

Conceptualization, BCh, PM, AZ and MM. Data curation, BCh and MM. Formal analysis, BCh. Funding acquisition, BCh and MM. Investigation, BCh, PM, AZ and MM. Methodology, BCh, AZ and MM. Project administration, BCh. Resources, BCh, PM, HV, and JD. Software, BCh, PM, TK, JL and MM. Supervision, PM, HV, JD and MM. Validation, BCh and MM. Visualization, MM. Writing – original draft, BCh and MM. Writing – review & editing, PM, AZ and MM. All authors contributed to the article and approved the submitted version.

## Funding

This work was granted by the Medical University of Bialystok. Poland (grant number: SUB/1/DN/21/002/1140; SUB/1/DN/21/002/3330; SUB/1/DN/21/002/1209; SUB/1/DN/22/002/3330.

## Conflict of interest

The authors declare that the research was conducted in the absence of any commercial or financial relationships that could be construed as a potential conflict of interest.

## Publisher’s note

All claims expressed in this article are solely those of the authors and do not necessarily represent those of their affiliated organizations, or those of the publisher, the editors and the reviewers. Any product that may be evaluated in this article, or claim that may be made by its manufacturer, is not guaranteed or endorsed by the publisher.
